# The improvement of motor symptoms in Huntington’s disease during cariprazine treatment

**DOI:** 10.1186/s13023-023-02930-z

**Published:** 2023-12-01

**Authors:** Reka Csehi, Viktor Molnar, Mariann Fedor, Vivien Zsumbera, Agnes Palasti, Karoly Acsai, Zoltan Grosz, Gyorgy Nemeth, Maria Judit Molnar

**Affiliations:** 1 Global Medical Division, Richter Gedeon Plc., Budapest, Hungary; 2 Institute of Genomic Medicine and Rare Disorders, Semmelweis University Budapest, Budapest, Hungary; 3 Eotvos Lorand Research Network-Semmelweis University Multiomics Neurodegeneration Research Group, Budapest, Hungary; 4 1428 Budapest Pf. 2, Üllői út 26., Budapest, 1085 Hungary

**Keywords:** Cariprazine, Vraylar, Reagila, Huntington’s disease, Motor function

## Abstract

**Background:**

Huntington’s disease (HD) is a progressive neurodegenerative disease, characterised by motor disturbances and non-motor (i.e., psychiatric) symptoms. Motor symptoms are the hallmark features of HD and take many forms. Their emergence is related to alterations in striatal dopaminergic neurotransmission: dopamine levels increase in the early stages of the disease, while more advanced stages are characterised by reduced dopamine levels. Such a biphasic change potentially explains the alterations in motor symptoms: increased dopamine-production induces hyperkinetic movements early in the disease course, while depleted dopamine storage leads to hypokinetic symptoms in the advanced phase. Dopamine D2-D3 partial agonists could be a promising treatment option in HD, as they have the potential to either elevate or lower the surrounding dopamine levels if the levels are too low or too high, respectively, potentially offering symptom-relief across the illness-course. Therefore, the present study aimed at exploring the effects of cariprazine, a dopamine D2-D3 partial agonist with high affinity to D3 receptors, on motor symptoms associated with HD.

**Methods:**

This was a single-centre, retrospective study where sixteen patients received off-label cariprazine treatment for 12 weeks (1.5-3 mg/day). Motor symptoms were evaluated using the Motor Assessment of the Unified Huntington’s Disease Rating Scale. Least Square (LS) Mean Changes from Baseline (BL) to Week 8 and Week 12 in the Total Motor Score (TMS) were analysed using the Mixed Model for Repeated Measures method. In addition, improvement from BL to Week 8 and 12 was calculated for all motor items.

**Results:**

Data of 16 patients were collected, but data of only 15 patients were analysed as one patient dropped out due to non-compliance. Significant changes were observed from BL to Week 8 (LS Mean Change: -9.4, p < 0.0001) and to Week 12 (LS Mean Change: -12.8, p < 0.0001) in the TMS. The improvement was captured in the majority of motor functions, excluding bradykinesia and gait. Mild akathisia was the most commonly reported side-effect, affecting 3 patients.

**Conclusion:**

This is the first study investigating the effectiveness of a D2-D3 partial agonist, cariprazine, in the treatment of HD. The findings of this study revealed that cariprazine was effective in the treatment of a wide range of motor symptoms associated with HD.

## Introduction

### Huntington’s disease

Huntington’s disease (HD) is a rare, progressive neurodegenerative disease with autosomal dominant inheritance [1]. It is caused by the expansion of a CAG triplet repeat within the huntingtin (IT15) gene, giving rise to an elongated polyglutamine tract in the resultant protein, therefore causing toxicity through a “gain-of-function” mechanism [2]. The most prominent clinical features include motor symptoms, cognitive impairment (e.g., dysfunction in executive functions, attention, learning and memory), and psychiatric alterations (e.g., depression, apathy, irritability, personality changes). The non-motor symptoms emerge early on and worsen progressively [1, 3]. The longer the polyglutamine repeat, the earlier symptoms start to manifest and the faster they progress. Age of onset ranges from childhood to the eighth decade, but symptoms most commonly appear in the fourth or fifth decades of life. Larger repeats (CAG > 55) are responsible for the juvenile form of HD (JHD), with symptoms appearing before the age of 20. In JHD, 42–94% of patients develop bradykinesia, rigidity, dystonia and psychiatric symptoms [4]. Approximately 30% of JHD patients present with psychiatric or behavioural disturbances (obsessive–compulsive behaviour) at onset [4].

By most, HD is considered predominantly a hyperkinetic movement disorder, as its most obvious and striking features are chorea and dystonia [5]. The early stage of the disease course is dominated by chorea, while dystonia and akinesia become dominant later on [1]. Oculomotor dysfunction can further be observed (e.g., supranuclear gaze palsy, choreatic eye movements) [5]. Despite non-motor symptoms often preceding the emergence of motor symptoms, they are rarely captured as the first signs of HD [6].

### Role of dopamine in Huntington’s disease

Dopamine is a major neurotransmitter playing an essential role in many centrally regulated functions, including attention, learning, memory, mood, motivation, reward and pleasure, motor functions, prolactin production and sleep [7, 8]. The dysregulation of the dopamine system is well-established in the majority of psychiatric and neurological disorders, including HD [9]. Among the five subtypes of dopamine receptors, D1, D2 and D3 play a major role in the pathophysiology of neuropsychiatric disorders and are therefore in the focus of research. Three of the four main dopamine pathways are involved in HD: the mesolimbic (connecting the ventral tegmental area to the ventral striatum), mesocortical (connecting the ventral tegmental area to the prefrontal cortex) and the nigrostriatal pathways (connecting the substantia nigra to the caudate and putamen; responsible for movement) [7].

The basal ganglia, consisting of the substantia nigra, globus pallidus, subthalamic nucleus and of particular importance for HD, the striatum (caudate nucleus and putamen), are a group of deep subcortical nuclei in the brain with extensive interconnections, and are responsible for motor control, cognition and emotion [10, 11].

Neuropathological alterations in HD mainly affect the striatum and the cerebral cortex [12]. Since 90–95% of striatal neurons are comprised of medium-sized spiny neurons (MSNs), the neurodegeneration of the striatum in HD results in a massive loss of these neurons [1, 13]. Two striatal projection pathways are differentiated: the direct (excitatory) and indirect (inhibitory) pathways.

The indirect pathway (Fig. 1A) is responsible for the suppression of undesirable movements and consists of MSNs that express D2 receptors [1]. The indirect pathway originates from the cortex, sending excitatory projections to the striatum, which then sends inhibitory projections to the external globus pallidus (GPe). The GPe provides inhibitory input to the subthalamic nucleus, which in turn projects excitatory input to the internal globus pallidus (GPi). The GPi is connected by an inhibitory loop to the thalamus, from where excitatory connection is established to the cortex. Therefore, the activation of the indirect pathway yields the increased inhibition of the thalamus and the cortex, resulting in movement-suppression [11].

**Fig. 1A:**
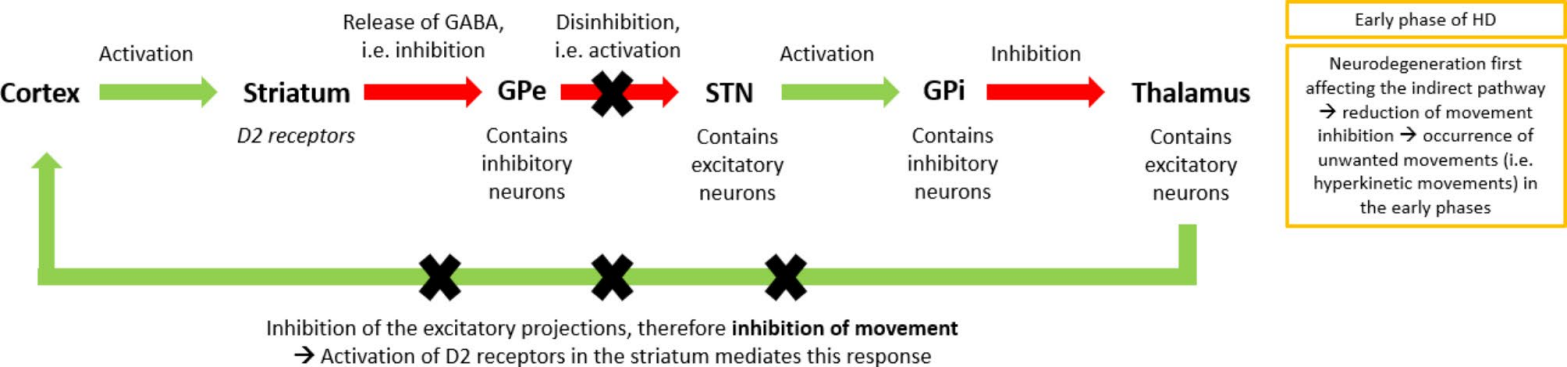
The indirect pathway and its disruption in HD

On the other hand, the direct pathway (Fig. 1B) has been associated with the control and initiation of voluntary movement, and consists of MSNs that express D1 receptors [11]. This pathway originates from the cortex, providing excitatory input to the striatum, from which the inhibitory projections terminate in the GPi. From the GPi, further inhibitory inputs are sent to the thalamus, while the thalamo-cortical projections are excitatory. Therefore, the activation of the MSNs in the direct pathway yields the disinhibition of the thalamus, which projects excitatory input to the cortex, initiating movement [11].

**Fig. 1B:**
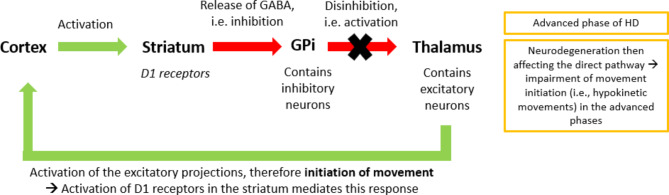
The direct pathway and its disruption in HD

In HD, neurodegeneration affects the MSNs of the indirect pathway early in the disease-course (Fig. 1A): as the number of these neurons decreases, the surplus glutamatergic and dopaminergic excitatory signals that would typically have been directed towards the indirect neurons are funnelled into the direct pathway. As the disease progresses, the direct pathway further becomes impacted by neurodegeneration (Fig. 1B). This biphasic pattern explains the sequence of the appearance of motor symptoms: hyperkinetic movements, such as chorea usually develop in the early phase due to impaired inhibition of motion control by the indirect pathway, while the subsequent impairment of the direct pathway results in hypokinetic state in the advanced stage [11, 14]. Therefore, having balanced dopamine levels is crucial for optimal motor performance: both high and low levels induce malfunction. Based on dopamine’s vital role in the motor symptoms of HD, compounds targeting the dopaminergic system could lead to improvements in motor function, especially dopamine partial agonists that can restore normal dopamine neurotransmission by either increasing or decreasing dopamine receptor activity depending on the amount of dopamine available in the synaptic cleft.

### Treatment of motor symptoms

There have been great efforts put into inventing causative treatments for HD, including the reduction of mutant huntingtin concentrations in the central nervous system via gene editing, gene therapy or antisense oligonucleotide approaches [15]. However, no curative or disease-modifying treatments are available yet, therefore symptom control provides the basis of disease-management [16].

The treatment of HD requires a multidisciplinary approach where the combinations of pharmacological and non-pharmacological treatment options are offered to patients tailored to their needs, even prior to the manifestations of symptoms. Pharmacological treatment (for a summary, see Table 1) differs for motor symptoms based on whether they are hyper- or hypokinetic [5]. Hyperkinetic manifestations are treated with medications targeting the dopaminergic system, like dopamine receptor antagonist antipsychotics targeting postsynaptic dopamine receptors, or tetrabenazine (TBZ), a reverse inhibitor of the vesicular monoamine transporter 2 (VMAT2), that concentrates dopamine within presynaptic vesicles [5]. TBZ, and a structurally related molecule with deuterium, deutetrabenazine (deuTBZ), have been shown to be efficacious in the reduction of hyperkinetic movements, such as chorea, dystonia or tardive dyskinesia [17]. However, the FIRST-HD study revealed that despite displaying similar efficacy, deuTBZ is associated with less side-effects compared to TBZ [18]. In line with these findings, a network analysis showed that TBZ was more likely to cause depression and somnolence than deuTBZ [17].

**Table 1:** Medications used to treat motor symptoms associated with HD

	Class	Name	Evidence
**Drugs approved by the FDA for HD**	VMAT2 inhibitor	Tetrabenazine	- Efficacious in reducing hyperkinetic movements - Associated with side-effects
		Deutetrabenazine	- Efficacious in reducing hyperkinetic movements - Associated with less side-effects than TBZ
**FDA-approved drugs, but not specifically for HD (off-label use)**	Antipsychotic	Aripiprazole	- Showed therapeutic potential in reducing chorea - No efficacy in improving cognition
		Haloperidol	- Some effectiveness in reducing chorea - Some effectiveness in reducing mutant huntingtin aggregate formation in rats - No improvement of functional capacity
		Risperidone	- Some effectiveness in improving motor symptoms
		Clozapine	- No effectiveness in reducing chorea (but controversial, probably on higher doses, but it increases side-effects)
	Dopaminergic stabiliser	Pridopidine	- In development for HD - Reduced UHDRS-modified motor score, but not TMS (only with higher doses, which caused more side-effects)

Antipsychotics acting on D2 receptors have demonstrated therapeutic potential as well [11]. Aripiprazole, a D2 receptor partial agonist, had similar efficacy inhibiting chorea as TBZ, although it failed to effectively improve cognition [19]. A D2 receptor antagonist, haloperidol, was shown to improve symptoms of chorea in some HD patients [20], as well as to reduce mutant huntingtin aggregate formation in a rat model of HD [21]. However, it did not yield an increase in functional capacity [22]. Furthermore, risperidone, a D2 receptor antagonist, showed superiority in the management of motor symptoms compared to placebo [23]. Clozapine could not effectively manage chorea, although results are controversial [24]. There is evidence that potentially higher doses of clozapine are required to achieve the desired effect on chorea [24], however, it may result in significant adverse effects, like fatigue, dizziness and gait disturbance [25]. In addition, a new dopaminergic stabiliser, pridopidine, was recently developed for the treatment of motor symptoms associated with HD. A systematic review and meta-analysis of four randomised controlled trials showed that pridopidine significantly outperformed placebo on the Unified Huntington’s Disease Rating Scale (UHDRS)-modified Motor Score (mMS), but not on the Total Motor Score (TMS) [26]. However, for a pridopidine dose of at least 90 mg/day, TMS also showed significant improvements in addition to the mMS, but also increased the occurrence of adverse events compared to placebo, such as nasopharyngitis and insomnia [26].

It is important to take into account that medications used in HD have the propensity to cause deteriorations in mood, cognition, and alertness. Therefore, it is crucial to consider the non-motor symptoms of HD as well when choosing a medication: the most optimal ones address all symptom domains of the disease, including mood, cognitive, psychiatric, and motor symptoms as well.

### Cariprazine

Cariprazine (CAR) is a dopamine D2-D3 partial agonist with preferential binding to the D3 receptors. It is approved for the treatment of schizophrenia by the European Medicines Agency [27] and by the Food and Drug Administration (FDA) [28], and for the treatment of depressive and manic/mixed episodes associated with bipolar disorder by the FDA. Furthermore, it has been recently approved as an adjunctive therapy in major depressive disorder by the FDA [29]. Cariprazine has a high affinity to D3, D2 and serotonin 5HT1A receptors at which it acts as a partial agonist, and to 5HT2B receptors, at which it acts as an antagonist [30]. Furthermore, it has a moderate affinity to serotonin 5HT2A and 5HT2C receptors, where it exerts antagonist activity [30]. It has two major metabolites, desmethyl CAR and didesmethyl CAR which are pharmacologically equipotent to CAR and they jointly achieve the overall therapeutic effect [31, 32].

### Study aims

The aim of the present study was to determine whether CAR is an effective pharmacological treatment option for controlling motor symptoms associated with HD.

## Methods

This study is a retrospective study aiming to investigate the efficacy of cariprazine in the treatment of motor symptoms in patients with Huntington’s disease. Non-motor symptoms (cognition and mood) were further evaluated, but the results are reported in another publication [33].

The Hungarian National Institute of Pharmacy and Nutrition granted permission for the off-label use of cariprazine for all participants. Ethical approval of the study was issued by the Regional, Institutional Scientific and Research Ethics Committee. The study was conducted in accordance with the Declaration of Helsinki, with the written informed consent of all participants.

Patients with an abnormal expansion in the huntingtin gene (CAG > 36) and with a clinical diagnosis according to the diagnostic confidence interval (ranges from 0 to 4) of the UHDRS were involved in the study. The diagnostic confidence level ranges from 0 (normal) to 4 (unequivocal extrapyramidal signs of HD, ≥ 99% confidence of the examiner).

The starting dose of cariprazine was 1.5 mg/day, which was increased to 3.0 mg/day, if deemed necessary. The use of co-medications, like TBZ, benzodiazepines, antidepressants or antipsychotics was allowed.

### Efficacy evaluation

To evaluate the motor symptoms, the UHDRS Motor Assessment subscale was administered, which consists of 15 items, each evaluated on a scale of 0 (normal) to 4 (severe). The TMS was calculated for all patients which indicated the severity of their motor symptoms. Evaluations were carried out by the same examiner at three different time-points: at the start of the treatment and at weeks 8 and 12.

Maximal chorea was examined in four different body regions (face; mouth; trunk; extremities).

For the assessment of maximal dystonia (tendency toward a posture, posturing along an axis), separate scores were given for symptoms in the trunk and in the extremities.

Movement coordination, rapid alternating movements and fine motor functions were evaluated with the Luria – fist-hand-palm sequencing; pronate/supinate hands; and the finger taps items.

In addition, bradykinesia, rigidity (based on the examination of the elbow and wrist), ocular pursuit (vertical and horizontal); saccade initiation (vertical and horizontal); saccade velocity (vertical and horizontal); dysarthria and tongue protrusion; gait; tandem walking; and retropulsion were evaluated.

### Safety evaluations

Safety evaluations were also conducted at baseline, Week 8 and Week 12. The assessments included body weight, vital signs, neurological examination, ECG, and routine laboratory testing, as well as assessments of motor function and adverse events.

### Statistical analysis

Mixed model for repeated measures (MMRM) was used to analyse each efficacy parameter separately, with the terms of visit, baseline parameter values and their interaction, assuming unstructured covariance structure and using Kenward-Roger’s approximation of the degrees of freedom. Least square (LS) mean changes were calculated and compared between visits. In addition, improvement from BL to Week 8 and 12 was calculated for all motor items, with improvements expressed in percentage.

## Results

### Patients

Patient demographics are summarised in Table 2. Altogether, 16 patients were enrolled in the study, but one dropped out due to non-compliance (Patient 4, who is therefore excluded from the Figures/Tables), thus the data of 15 patients were analysed: 4 males and 11 females. Patients had a mean age of 48.13 years (SD = 10.60) and mean disease duration of 3.79 years (SD = 2.89).

**Table 2:** Patient demographics

Participant	Sex	Age	Disease duration	TFC	CAR dose (mg/day)	Tetrabenazine dose (mg)
**P1**	M	42	4	10	1.5	2 × 25
**P2**	F	48	1	10	1.5	3 × 25
**P3**	F	51	4	10	1.5	3 × 7.5
**P5**	F	50	6	12	3	
**P6**	F	36	6	10	1.5	4 × 25
**P7**	F	40	0.5	15	4.5	
**P8**	F	53	1	5	1.5	2 × 12.5
**P9**	M	74	8	6	1.5	3 × 25
**P10**	F	43	1	6	1.5	
**P11**	M	55	10	1	1.5	4 × 25
**P12**	F	42	1	10	1.5	3 × 12.5
**P13**	F	66	4	8	1.5	3 × 7.5
**P14**	F	43	4	5	1.5	3 × 50
**P15**	M	42	2	12	1.5	
**P16**	F	37	1	12	1.5	

Regarding cariprazine doses, 13 patients received 1.5 mg/day, one received 3.0 mg/day, and another received 4.5 mg/day. Ten patients were taking a stable dose of tetrabenazine during the treatment period.

### Safety evaluations

Three patients reported side-effects of cariprazine: two of them developed akathisia, and one experienced akathisia with weight loss.

### Efficacy evaluations

**Fig. 2:**
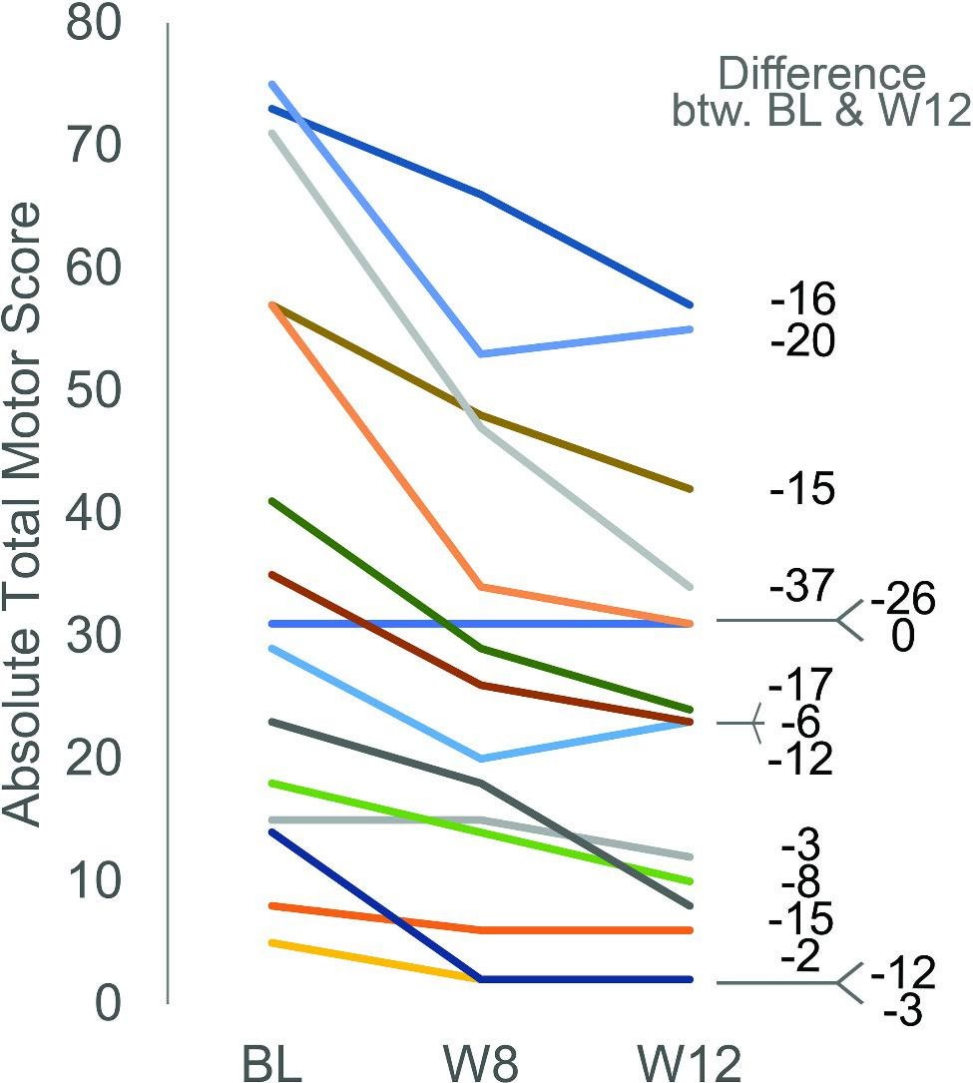
Individual courses of motor symptom development during cariprazine treatment Absolute values of the UHDRS motor scores are shown BL: baseline; W8: week 8; W12: week 12

Mean TMS score at the start of the treatment was 36.8, which decreased to a mean score of 27.4 at week 8 [least square (LS) mean change: -9.4, p < 0.0001; 26% improvement] and to 24.0 at week 12 (LS mean change: -12.8, p < 0.0001; additional 12% improvement) (Fig. 2; Table 3).

**Table:** Individual baseline TMS scores (in bold) and the differences between BL values and week 8 or 12, respectively P: participant

	Baseline	Week 8	Week 12
P1	**15**	0	-3
P2	**35**	-9	-12
P3	**31**	0	0
P5	**8**	-2	-2
P6	**75**	-22	-20
P7	**5**	-3	-3
P8	**18**	-4	-8
P9	**57**	-9	-15
P10	**41**	-12	-17
P11	**73**	-7	-16
P12	**29**	-9	-6
P13	**57**	-23	-26
P14	**71**	-24	-37
P15	**23**	-5	-15
P16	**14**	-12	-12

On the maximal chorea measure, patients showed significant improvements: symptoms decreased by 35% at Week 8 and by 52% at Week 12 compared to BL. Table 4 and Table 5 show the individual scores on different motor subscales at BL, Week 8 and Week 12.

The alleviation of (maximal) dystonia was further observed: from baseline, symptoms improved by 57% at Week 8 and by 85% at Week 12 (Table 4, Table 5).

Significant improvements were observed on the Luria; pronate/supinate hand; and the finger taps measures, indicating that the treatment positively impacted on the hand movements. Compared to BL, on Luria’s test, patients showed a 22% improvement at Week 8 and a 19% improvement at Week 12; in finger tapping, patients exhibited a 9% improvement at Week 8 and a 26% improvement at Week 12. On the pronate/supinate test, no effects were observed at Week 8, however at Week 12, patients showed a 9% improvement (Table 4, Table 5).

Regarding bradykinesia, 5 patients had slight worsening by Week 12. Rigidity (arms) of the elbow and wrist joints affected three patients at BL and by Week 12, it resolved completely, while one patient developed rigidity by Week 12 (Table 4, Table 5).

Improvements in oropharyngeal symptoms were observed. Mean score for dysarthria reduced from 1.07 to 0.87, signalising a 19% improvement. This is further evidenced by the 28% improvement in tongue protrusion at Week 12 (Table 4, Table 5).

Although no objective change was observed in patients’ gait, they reported to walk more stable. Regarding tandem gait, a 25% average improvement was noted and the retropulsion pull test showed a 39% improvement by Week 8. Although the improvement fell back to 29% by Week 12, the improvement is considered significant (Table 4, Table 5).

Regarding voluntary eye movements, improvements were detected on all three measures. Overall, eye movements improved by 35% by Week 8 and by 47% by Week 12, compared to BL. (Table 4, Table 5).

The diagnostic confidence level did not change, neither at Week 8 nor at Week 12.

**Table 4:**
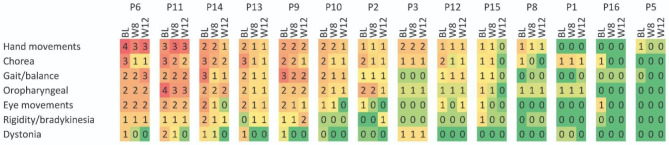
Heat map illustrating the individual severity of the different UHDRS domains at BL, Week 8 and Week 12 The items of UHDRS motor scale were summarised in subscales (sum of corresponding values and divided by the number of questions regarding the problem) by preserving the range between 0 and 4. Patients in columns and subscales in rows were ranked in descending order according to the sum of baseline values among patients and subscales, respectively (On the two heatmaps the patients in columns follow the same order for comparison). The highest (2<=) and virtually lacking (0,1>) cells were highlighted for better comparison of the two time points 0: no symptoms; 1: mild symptoms; 2: moderate symptoms; 3: moderately severe; 4: severe symptoms BL: baseline; W8: week 8; W12: W12; P: participant

**Table 5:** Individual scores on different motor subscales at BL, Week 8 and Week 12 BL: baseline; W8: week 8; W12: W12; P: participant

	P1			P2			P3			P5			P6			P7			P8			P9			P10			P11			P12			P13			P14			P15			P16		
	BL	W8	W12	BL	W8	W12	BL	W8	W12	BL	W8	W12	BL	W8	W12	BL	W8	W12	BL	W8	W12	BL	W8	W12	BL	W8	W12	BL	W8	W12	BL	W8	W12	BL	W8	W12	BL	W8	W12	BL	W8	W12	BL	W8	W12
**Hand movements**	0	0	0	7	5	6	8	8	8	3	2	2	18	15	17	2	1	1	7	4	3	11	11	9	9	8	7	13	17	16	7	6	6	9	7	6	12	11	7	5	4	2	0	0	0
**Chorea**	8	8	6	13	8	7	9	9	9	0	0	0	20	7	6	0	0	0	6	3	0	16	13	8	12	7	7	20	16	11	11	4	5	18	10	7	18	14	11	5	6	1	5	0	0
**Gait/balance**	0	0	1	2	2	2	1	1	1	1	0	0	6	6	8	2	0	0	0	1	1	9	7	7	5	4	4	6	7	7	2	3	3	5	3	3	9	3	4	2	2	1	1	1	1
**Oropharyngeal**	1	1	1	4	4	2	1	1	1	0	0	0	4	4	4	0	0	0	1	1	1	3	3	3	3	2	2	7	5	5	1	1	1	3	2	2	4	3	4	2	1	0	0	0	0
**Eye movements**	0	0	0	4	2	0	2	2	2	0	0	0	12	12	12	0	0	0	0	0	0	9	8	6	6	4	0	12	10	10	4	2	4	10	6	7	14	6	2	4	2	1	6	0	0
**Rigidity/bradykinesia**	0	0	0	0	0	2	1	1	1	0	0	0	4	4	4	0	0	0	0	1	1	2	2	5	1	1	1	3	3	3	1	1	1	1	2	2	5	3	2	3	1	1	1	0	0
**Dystonia**	2	2	0	1	1	0	5	5	5	0	0	0	7	2	0	0	0	0	0	0	0	3	0	0	2	0	0	8	4	1	0	0	0	7	0	0	5	3	0	0	0	0	0	0	0

## Discussion

This is the first study investigating the effect of a D2-D3 partial agonist in the treatment of HD, which is a neurodegenerative disease characterised by dopamine imbalance. The findings of this study show that cariprazine was effective in the treatment of a wide range of motor symptoms associated with HD.

Cariprazine’s efficacy in the treatment of the motor symptoms of Huntington’s disease could be explained by its high affinity to D3 receptors. In fact, cariprazine’s distinctive characteristic lies in that it is the only approved antipsychotic with proven in-vivo D3 affinity, as demonstrated by PET studies - its affinity to D3 receptors is even greater than that of dopamine itself [30, 34]. This makes cariprazine the only drug that can occupy the D3 receptors in the presence of dopamine in vivo [35]. The role of the D3 receptors have been implied, and there are some potential mechanisms via which D3 receptor activity yields improvements in motor symptoms.

In HD patients, the most significant neurodegeneration occurs in the caudate and putamen – brain areas containing high levels of dopamine receptors that are involved in motor function [36]. The dopamine D2 and D3 receptors are highly expressed in midbrain dopaminergic neurons, MSNs of the striatum and in diverse neuronal populations in the cerebral cortex [37]. Therefore, given the atrophy of the MSNs in the striatum [38], molecules targeting the D2-D3 receptors could be a treatment option for the motor symptoms associated with HD, and cariprazine’s efficacy could potentially be attributed to this due to its high affinity to both receptors.

Another notable fact is that D3 receptors synergistically promote the biological effects of D1 receptor stimulation [39]. It is noteworthy that the D3 receptors are co-localised with D1 receptors in the striatum, implying that the regulation of MSN function might partly be attributed to the joined activity of these two receptor systems [40, 41]. The co-localisation of D3 and D1 receptors allows them to form heteromers, resulting in functional integration. Via synergistic interaction, D3 receptor activation might enhance the affinity of dopamine [40, 42, 43], which in turn increases the D1-mediated transmission within the D1-D3 heteromers. Given the reduction in D1 receptor expression in the striatum in HD, its stimulation could alleviate motor symptoms via the D3 receptors, therefore cariprazine might facilitate this mechanism.

Growing evidence suggests that D3 stimulation has neurotrophic, neuroprotective and neurorestorative effects on dopamine neurons. Therefore, D3 receptors might have an essential role in preventing pathological alterations underlying neurodegeneration [40]. The activation of the D3 receptors has been shown to facilitate neurogenesis - interestingly, with some studies indicating that adult neurogenesis also occurs in the striatum [39, 44]. Therefore, it is possible that prolonged exposure to D3 receptor activation would contribute to neurogenesis and therefore motor improvement in patients with HD.

The most optimal treatment of HD is one that addresses the wide-ranging symptoms of the disease – i.e., not only the motor, but the cognitive, mood and other psychiatric symptoms as well. Although TBZ and deuTBZ are the only approved treatments for HD chorea, they have been shown to worsen cognition and mood. Since these symptoms are associated with worse patient functionality and quality of life, adequately addressing them is of utmost importance.

In addition to cariprazine being effective in the improvement of motor symptoms, as seen in the present study, it has been shown to improve non-motor symptoms as well [33]. The paper by Molnar and colleagues reported on the same study as this current paper, but reporting on the outcomes related to non-motor symptoms only. They showed that cariprazine yielded significant improvement in mood and behavioural symptoms, as well as in cognition. Cariprazine’s efficacy in cognition [45] and mood [28, 29, 46] was observed in other neuropsychiatric disorders, like schizophrenia, bipolar disorder and major depressive disorder.

Regarding the safety evaluations in this patient population, akathisia was the most commonly reported side-effect, which is in line with previous findings [27]. Akathisia can be managed either by reducing the dose of cariprazine or by adding medications, like beta-blockers, to alleviate this side effect. These strategies were used in schizophrenia patients in clinical trials, as well as they are recommended by experts [47, 48]. In the trials, 85% of akathisia events resolved within a median of 17 days after the administration of an anti-akathisia medication. In case of down-titration of cariprazine, akathisia resolved within a median of 15 days in over 90% of events. Therefore, it is recommended to try one of these strategies before withdrawing cariprazine.

The other reported side effect was weight loss, present in only one of the patients. Cariprazine is a metabolically neutral medication causing little to no weight gain, therefore it is in line with previous findings [47]. However, since weight loss is a common feature in HD, there is no clear evidence that this event was solely associated with cariprazine, it could be a symptom of the disease per se.

Although cariprazine was effective in reducing motor symptoms associated with HD without causing any serious adverse events, a noteworthy observation needs to be addressed: when analysing the individual data, a slight regression in improvement in the UHDRS motor score can be observed in a few cases (see Fig. 2): three patients stagnated at Week 12 compared to Week 8 (i.e. no further improvement; Patients 5, 7 and 16), while two patients experienced a slight increase in their motor score at Week 12 compared to Week 8 (Patients 6 and 12). There are some potential explanations for this observation. Firstly, after investigating the demographic data of these patients (compared to others), it is apparent that these patients were in the early stage of the disease and they had high TMS score indicating high functionality as well as relatively low motor scores at baseline (except for Patient 6), indicating less severe motor symptoms. Therefore, at the time of the study, these patients were not so heavily impacted by the disease yet, thus lesser improvement/stagnation after an initial improvement can be expected. This was confirmed by the regression plot that was further generated, showing that patients with more severe symptoms at baseline experienced greater improvements during the treatment period. The second explanation for the slight regression in improvement is that motor symptoms can naturally show slight, spontaneous fluctuations. Evaluating such symptoms at given timepoints can result in sometimes “unexpected” observations. Lastly, medication non-compliance can further contribute to improvement-regression. Although the examiners monitored potential non-compliance and excluded patients from the analysis in such cases (like Patient 4), of course this cannot be ruled out completely.

The present study holds some limitations. Firstly, given the retrospective design of the study and the lack of control arm, causality cannot be drawn and further research with more rigorous design and control arm is warranted in order to confirm the effectiveness of cariprazine in motor symptoms associated with HD. However, since no trials have investigated cariprazine in HD before, these preliminary findings provide a great foundation and rationale for future research. Next, the sample size is considered to be relatively small, however, since HD is a rare disease, it makes it difficult to recruit a large number of patients – nonetheless, studies should aim for including more patients to improve the generalisability of the findings.

In conclusion, our findings indicate that cariprazine is a promising medication that has the potential to alleviate both motor and non-motor [33] symptoms of HD while being well-tolerated by patients.

## Data Availability

All data and material are available in the Institute of Genomic Medicine and Rare Disorders.
